# Complete genome sequence of *Edwardsiella* sp. NBRC12716 isolated in 1962 from the liver of diseased eel

**DOI:** 10.1128/MRA.00737-23

**Published:** 2023-09-29

**Authors:** Andre Lanza, Hideaki Mizobata, Ryo Yonezawa, Kazutoshi Yoshitake, Kinoshita Shigeharu, Shuichi Asakawa

**Affiliations:** 1 Department of Aquatic Bioscience, Graduate School of Agricultural and Life Sciences, The University of Tokyo, Tokyo, Japan; University of Southern California, Los Angeles, California, USA

**Keywords:** fish pathogens, Nanopore sequencing, long-read sequencing, *Edwardsiella*, pangenomics

## Abstract

We report the complete genome sequence of *Edwardsiella* sp. NBRC12716 isolated from a diseased eel in 1962. The genome consists of a single, circular chromosome 3,771,060 bp in length with 59.74% GC content and encodes 25 rRNA, 96 tRNA, and 3,182 protein-coding genes.

## ANNOUNCEMENT

The genus *Edwardsiella* includes several species that are notorious pathogens of farmed and wild fish with overlapping host ranges (1). Historically, this obscured their classification (2–7), and several fish isolates collected decades ago and stored in biological repositories remain unclassified. Genome sequencing of these isolates can aid their classification and expand the pangenome sequence space for *Edwardsiella* allowing for detailed study of these pathogens, thus yielding better insights into their control and treatment. Here, we report the complete genome sequence of an unclassified *Edwardsiella* sp. strain, NBRC12716, isolated from the liver of diseased eel (*Anguilla japonica*) in 1962 in Japan.

The NBRC12716 16S rRNA gene was sequenced by the NITE Biological Resource Center (NBRC) in 2011 (AB680333.1). A lyophilized sample was obtained from NBRC and revived on tryptic soy agar (Millipore) at 30°C overnight. The following day, a single colony was inoculated in tryptic soy broth (ForMedium) and incubated at 30°C on a shaker for 16 h. DNA was extracted using a Nanobind Tissue Big DNA Kit (Circulomics), quantified on a Qubit v2.0 fluorometer (ThermoFisher Scientific), and quality checked on a TapeStation 2200 System (Agilent) (DNA integrity number, 9.8). DNA was not size selected prior to sequencing. Sequencing libraries were prepared using a Ligation Sequencing Kit [SQK-LSK110; Oxford Nanopore Technologies (ONT)] and sequenced on a FLO-FLG001 flow cell (R9.4.1) (ONT) on the MinION platform. MinKNOW (v23.04.3) was used to collect signal data in POD5 files (https://github.com/nanoporetech/pod5-file-format), and bases were called with Dorado (v0.3.0) (https://github.com/nanoporetech/dorado) in super accurate mode (dna_r9.4.1_e8_sup@v3.6). NanoLyse (8) (v1.2.1) and PoreChop ABI (9) (v0.5.0) were used to remove lambda and adapter sequences, respectively. NanoFilt (8) (v2.8.0) was used to trim 50 bp at both ends of the reads, and reads with mean *Q* scores greater than 8 and 12 were retained separately. Q8 reads were assembled using Flye (10) (v2.9.2) (estimated genome size: 4 Mbp), and the assembly was confirmed to be circular (by Flye). The primary assembly was then polished using Q12 reads with Medaka (v1.5.0) (model: r941_min_sup_g507) and rotated to start with the *dnaA* gene by Circlator (11) (v1.5.5). CheckM (12) (v1.2.2) was used to assess the assembly completeness and contamination, and fastANI (13) (v1.33) was used to compute the average nucleotide identity (ANI) in comparison to reference genomes of representative *Edwardsiella* spp. Gene annotation was conducted with the NCBI Prokaryotic Genome Annotation Pipeline (14) (v6.5). Default parameters were used for all software unless noted otherwise.

The *Edwardsiella* sp. NBRC12716 genome consists of a single, circular chromosome 3,771,060 bp in length with a GC content of 59.74% (Table 1). It encodes 25 rRNA, 96 tRNA, and 3,182 protein-coding genes. CheckM reported a completeness of 90.22% and contamination rate of 2.73%. NBRC12716 showed the highest ANI with *Edwardsiella piscicida* 18EpOKYJ (GCF_021733145.1) at 99.9% followed by *Edwardsiella anguillarum* ET080813 (GCF_000264765.2), *Edwardsiella ictaluri* S07-698 (GCF_003074995.2), *Edwardsiella tarda* KC-Pc-HB1 (GCF_002504285.1), and *Edwardsiella hoshinae* FDAARGOS_940 (GCF_016026395.1) at 95.0%, 92.5%, 84.1%, and 83.6%, respectively, suggesting its reclassification to *E. piscicida*. Pangenomic analysis of this and other *Edwardsiella* strains will bolster our understanding of the pathogenic species which can guide biosecurity measures against them.

**TABLE 1:** Nanopore sequencing and genome assembly statistics of *Edwardsiella* sp. NBRC12716

Strain	Bases called (Mbp)	No. of basecalled reads	Read N_50_ (kb)	No. of contigs	Total genome length (bp)	Coverage (×)	GC content (%)	CheckM statistics	
								Completeness (%)	Contamination (%)
NBRC12716	511.30	51,867	29.88	1	3,771,060	136	59.74	90.22	2.74

## Data Availability

This Whole Genome Shotgun project has been deposited to NCBI under the BioProject no. PRJNA1001143 with BioSample no. SAMN36795961 and accession no. CP131923. Raw sequences can be accessed at SRR25514213.
